# Wide circulation of peste des petits ruminants virus in sheep and goats across Nigeria

**DOI:** 10.4102/ojvr.v88i1.1899

**Published:** 2021-09-07

**Authors:** Samuel E. Mantip, Anthony Sigismeau, Maurice Nanven, Atuman Joel, Abayomi M. Qasim, Sada Aliyu, Ibrahim Musa, Ogechukwu Ezeanyika, Ibikunle Faramade, Garba Ahmed, Timothy Y. Woma, David Shamaki, Genevieve Libeau, Souaibou Farougou, Arnaud Bataille

**Affiliations:** 1 Communicable Disease Research Unit, Polytechnic School of Abomey-Calavi, University of Abomey-Calavi, Contonou, Benin; 2 Virology Division, National Veterinary Research Institute, Vom, Nigeria; 3 CIRAD, UMR ASTRE, Montpellier, France; 4 ASTRE, University Montpellier, CIRAD, INRA, Montpellier, France

**Keywords:** Peste des petits ruminants, Nigeria, transboundary animal disease, virus isolation, RT-PCR, morbillivirus, sequencing

## Abstract

Peste des petits ruminants (PPR) is a highly contagious viral disease that mainly affects goats and sheep in Asia, Africa and the Middle East, and threatens Europe [R.E.1]. The disease is endemic on the African continent, particularly in West Africa, and is a major factor driving food insecurity in low-income populations. The aim of this research study was to carry out surveillance, genetic characterisation and isolation of recently circulating PPR viruses (PPRV) in sheep and goats from the six agro-ecological zones of Nigeria. A total of 268 post-mortem tissue samples of lung and mesenteric ganglia were collected from clinically suspected sheep and goats in 18 different states, of which five never previously sampled. The presence of PPRV was confirmed using a reverse-transcription coupled with a polymerase chain reaction (RT-PCR) assay. A total of 72 samples, 17 sheep (6%) and 55 goats (21%), were found to be PPR positive. Positive samples were distributed in almost all states, except Kano, where PPR was detected in previous studies. The PPRV-positive samples were further confirmed by sequencing or virus isolation in areas where the infection had never previously been detected. These results confirm the active circulation of PPRV across all six agro-ecological zones of Nigeria, and consequently, the need for introducing strict measures for the control and prevention of the disease in the country.

## Introduction

Peste des petits ruminants (PPR) is an acute, highly contagious viral disease mainly affecting goats and sheep. The disease was first reported in 1942 by Gargadennec and Lalanne (1942) in sheep and goats that resembled rinderpest in the West African country of Ivory Coast. However, the PPR virus (PPRV) was not isolated until 1967 (Bourdin & Laurent-Vautier 1968). The PPRV belongs to the family Paramyxoviridae and genus *Morbillivirus* (Gibbs et al. 1979), with taxonomic name *Small ruminant morbillivirus* (Amarsingh et al. 2019). This genus currently includes seven known members: measles virus, PPRV, canine distemper virus, seal distemper virus, cetacean morbillivirus, feline morbillivirus and rinderpest virus. In addition to the endemic presence of PPR in sub-Saharan Africa in the past decades, in recent years, field data and laboratory findings have confirmed the dramatic southward spread of PPR, affecting Gabon, the Democratic Republic of Congo, Somalia, Kenya and Tanzania (Swai et al. 2009). An outbreak of PPR infection was reported in Angola for the first time in October 2012 and in Zambia in July 2015 (Baron et al. 2016). The risk of introduction of PPR is now high in neighbouring countries with major sheep and goat populations, such as the Republic of South Africa and Mozambique. In addition to Africa, PPR infection has been reported in many Asian countries, including China. After an initial identification in Tibet in 2007 (Wang et al. 2009), a major PPR epizootic was reported there in 2013–2014 (Bao et al. 2017). As PPR has an economic impact, it is on the list of notifiable diseases to the International Office of Epizootics (OIE, World Animal Health Organization, https://www.oie.int) in the event of emergence. It is now the subject of a worldwide eradication campaign by OIE and FAO-UN (Food and Agriculture Organization of the United Nations; FAO & OIE 2015). PPRV transmission is only horizontal, by contact, which can be direct or indirect. Indirect transmission of the virus can be through ingestion of food or contaminated water or aerosolised transmission (Libeau et al. 2014). Goats are usually more sensitive than sheep, which have a higher recovery rate (Khan et al. 2009). Symptoms include fever, oculo-nasal discharge, diarrhea, dyspnoea, and effusion of the epithelium of the oral and nasal mucous membranes. Nasal and ocular discharges become mucopurulent, which causes a faint odour (Wohlsein & Saliki 2006). The rate of PPRV-related morbidity in a flock can reach 100%, and mortality is estimated at 90% (Singh et al. 2009). PPRV is classified into four genetically distinct lines, ‘lineage I’ to ‘lineage IV’, and all four are currently circulating in Africa (Mantip et al. 2016).

In Nigeria, sheep and goats are widespread across different ecological and climatic zones of the country. It is estimated that these animals provide more than 35% of total animal protein consumption in the country (Mantip et al. 2016). The total industrial value of small ruminants in Nigeria is around 40 billion naira (~€100 million (euros), Shamaki 2002). The results obtained in previous studies suggest that PPR remains an endemic disease in the country, accompanied by sporadic epidemics. Strains of PPRV of both lineages II and IV have been found to circulate in Nigeria in 2012. However, areas where the disease was considered as being exotic in the past could be the places today of regular epidemics because of an increase in trade and commerce. The aim of this research study was to provide an update on PPR circulation and epidemiology in sheep and goats across Nigeria using molecular diagnostics and virus isolation.

## Materials and methods

### Study area

**FIGURE 1:**
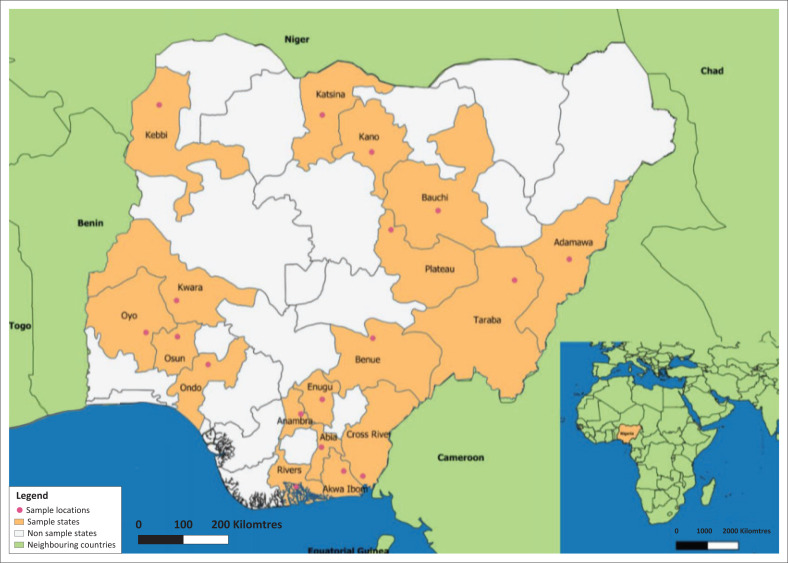
Sampling locations in different states of Nigeria over the period 2017–2018.

The study area included all six agro-ecological zones of Nigeria: north-central, north-west, north-east, south-west, south-east and south-south. In order to obtain a representative sample of sheep and goat flocks in the country, samples were collected in three states in each agro-ecological zone. Sampling was conducted between 2017 and 2018 in 18 states: Bauchi, Adamawa, Taraba, Kano, Kebbi, Katsina, Plateau, Kwara, Benue, Oyo, Osun, Ogun, Abia, Anambra, Enugu, Akwa Ibom, Cross River and Rivers (Figure 1 and Table 1).

**TABLE 1:** List of Peste des petits ruminants viruses-positive samples and partial N gene sequences obtained during this study.

Location	Species	Sample	N	Year	Accession number
**North-central zone**					
Plateau state	Goat	Lg, Ln	8/20	2017–2018	-
Benue state	Goat	Lg, Ln	2/20	2018	-
Kwara state	Goat	Lg, Ln	3/17	2017–2018	-
	Sheep	Lg, Ln	0/3	2017–2018	-
**North-east zone**					
Bauchi state	Goat	Lg, Ln	4/15	2017–2018	-
	Sheep	Lg, Ln	1/5	2017–2018	-
Adamawa state	Goat	Lg, Ln	0/12	2017–2018	-
	Sheep	Lg, Ln	1/8	2017–2018	-
Taraba state	Goat	Lg, Ln	2/14	2017–2018	-
	Sheep	Lg, Ln	0/6	2017–2018	-
**North-west zone**					
Kano state	Goat	Lg, Ln	0/14	2017–2018	-
	Sheep	Lg, Ln	0/6	2017–2018	-
Katsina state	Goat	Lg, Ln	3/13	2017–2018	MT193237
	Sheep	Lg, Ln	1/7	2017–2018	-
Kebbi state	Goat	Lg, Ln	5/6	2018	MT193247
	Sheep	Lg, Ln	7/14	2018	-
**South-east zone**					
Enugu state	Goat	Lg, Ln	2/11	2018	MT193249
	Sheep	Lg, Ln	1/9	2018	-
Abia state	Goat	Lg, Ln	3/13	2018	MT193250
	Sheep	Lg, Ln	4/7	2018	-
Anambra state	Goat	Lg, Ln	6/13	2018	-
	Sheep	Lg, Ln	3/7	2018	-
**South-west zone**					
Oyo state	Goat	Lg, Ln	5/20	2018	-
Ondo state	Goat	Lg, Ln	1/20	2018	-
Osun state	Goat	Lg, Ln	3/20	2018	-
**South-south zone**					
Rivers state	Goat	Lg, Ln	1/20	2018	MT193235
Cross-rivers state	Goat	Lg, Ln	2/20	2018	-
Akwa-Ibom state	Goat	Lg, Ln	4/20	2018	-

**Total**	**-**	**-**	**73/360**	**-**	**22**

Note: Location is the town where the samples were collected or the main town closest to the sampling site. Type indicates if samples were collected from a flock or in a market. *N* is the number of positive samples or total samples tested. Accession number, GenBank accession number. Multiple accession numbers are given for one location when multiple non-identical sequences were obtained from PPRV-positive samples. Accession numbers are repeated when the same sequence was obtained from several locations. The total accession number refers to the total number of non-identical sequences obtained.

Lg, lung; Ln, lymph node.

Sampling was carried out in each of these states by moving from one market place to another, with markets having been judged to be the most important location for sampling in each state. Target sampling size was 60 samples per zone and 20 locations per state. The sampling location was mapped using GPS coordinates. Goats and sheep suspected of being infected by PPR were identified based on clinical symptoms, which included fever over 40 °C, weight loss, ocular-nasal discharge and diarrhea. Veterinarians belonging to the Nigerian national veterinary services conducted the field studies in accordance with local legislation. The suspected animals were purchased from their owners, systematically euthanised, and an autopsy was performed to collect tissue samples. In addition, samples were collected from slaughterhouses located near the markets. The tissue samples collected from most animals had visible traces of diarrhea and oculo-nasal discharges on their carcasses. The tissue samples were immediately packed with ice and ice accumulators, and transported to the PPR laboratory of the National Veterinary Research Institute, Vom, Plateau State, Nigeria, and stored in a freezer at –70 °C before the sampling team moved on to another state. The samples were subsequently shipped to CIRAD, Montpellier, France for further laboratory analyses.

### Molecular detection of the virus nucleic acid

Tissue samples were cut into pieces and ground in 3 mL of Minimum Essential Medium (MEM) by vortexing with 0.2 µm glass beads. The swabs were placed in 1 mL MEM and vortexed. In all cases, the sample suspensions were centrifuged for 3 min at 1000 g and the supernatant was collected. Total ribonucleic acid (RNA) was extracted with an extraction robot KingFisher^TM^ and ID Kit Gene^TM^ Mag Universal Extraction (IDvet, France), as described by the manufacturer. The molecular diagnosis of PPR for each sample was performed by reverse-transcription coupled with a polymerisation chain reaction (RT-PCR) that uses the pair of primers NP3 (5’-GTC-TCG-GAA-ATC-GCC-TCA-CAG-ACT-3’) and NP4 (5’-CCT-CCT-CCT-GGT-CCT-CCA-GAA-TCT-3’) that specifically target the partial N gene (Couacy-Hymann et al. 2002). The RT-PCR was performed using Quantabio qTXT XLT One-Step RT-PCR kit, following manufacturer’s instruction. The RT-PCR mixture (25 µL) was composed of 5 µL RNA, 12.5 µL of One-Step Tough-Mix (2X), 1.5 µL of NP3 10 µM, 1.5 µL of NP4 10 µM, 1 µL of qScript XLT 1-Step RT 25X, 3.5 µL of H_2_O and 5 µL of nucleic acids from each sample. The RT-PCR cycle programme consisted of RT for 20 min at 48 °C, denaturation for 3 min at 94 °C; 40 cycles of 15 s at 94 °C, 30 s at 60 °C and final extension for 1 min at 72 °C.

The presence of PCR product of the expected size (355 bp) was controlled by 1.5% agarose gel migration. The fragment size of our amplicon was monitored using a 100-bp molecular weight marker after illumination under UV radiation or blue light.

### Phylogenetic analysis

When positive PCR products were obtained from areas where PPR had never previously been detected (Kebbi, Katsina, Rivers, Enugu and Abia states; see the Results section), one positive sample per state was selected based on the brightness of the amplicon on the gel, and sent to GENEWIZ (United Kingdom) for purification and sequencing in both forward and reverse directions. The sequences were submitted to the NCBI GenBank database (Table 1). Forward and reverse deoxyribonucleic acid (DNA) sequences were assembled using BioEdit and trimmed to remove poor-quality portions of the sequences (final size = 255 base pairs [bp]). Corrected sequences were aligned with a dataset of PPRV N gene sequences publicly available in GenBank using MEGA 6 representatives of the four genetic lineages.

The phylogenetic tree was constructed using the maximum likelihood method implemented in MEGA 6, with node supports evaluated by bootstrap analyses with 1000 replicates.

### Cell culture and virus isolation

Virus was isolated from field samples on canine histiocytic sarcoma (CHS) cells using the same five PCR positive samples sent for sequencing. CHS cells are CV1 cells (monkey cell line) modified to express SLAM receptors (‘Signaling Lymphocytic Activation Molecule’), or CD150 of goats, which are highly sensitive to PPRV growth (Adombi et al. 2011). These cells, which are very sensitive to PPRV infection, were used to isolate the virus from field samples. Five samples from different states that showed strong PCR amplification results were selected for the isolation attempt. Aliquots (150 mL) of viral solution contained in the homogenate samples were inoculated into the CHS cell mat seeded at a minimum confluence of 70% in 25 cm^2^ flasks.

The flask was gently shaken at 15-min intervals for 1 h. Culture medium was composed of MEM, foetal calf serum (FCS), an antibiotic and antimycotic was added. Antibiotics and antimycotics help to protect cells from potential contamination by pathogens (bacteria, fungi, etc.) that may be present in the sample. The infected flasks were incubated with 5% CO_2_ at 37 °C. The cytopathogenic effect (CPE) occurs after varying intervals because of the different viral loads in the samples. Incubation was stopped when the CPE reached 80% (Figure 3), and the flasks were subjected to cycles of freezing (–80 °C) and thawing (at room temperature or ambient temperature). These cycles cause the cells to burst and release viral particles. Culture medium that contained dead cells and viral particles was collected and centrifuged. The supernatant of the culture medium was used to extract the total nucleic acids as explained above.

Some samples showed bacterial or fungal contamination during the first isolation attempt. A second attempt, including an additional filtration step and the addition of a second antibiotic, was, therefore, necessary. The size of the filter pores used was 0.45 mm. The bacteria are all retained by pores of this size. The second antibiotic used was gentamycin. Subsequently, the presence of the virus in the medium was confirmed by collecting and testing the cell culture supernatant by RT-PCR.

### Ethical considerations

Approval to conduct the study was received from the Ethics Committee: National Veterinary Research Institute, Animal Use and Care Committee (AUCC, NVRI, Vom; reference No. AEC/02/87/20). Approval was also received from the University of Abomey-Calavi, Benin Republic.

## Results

**FIGURE 2:**
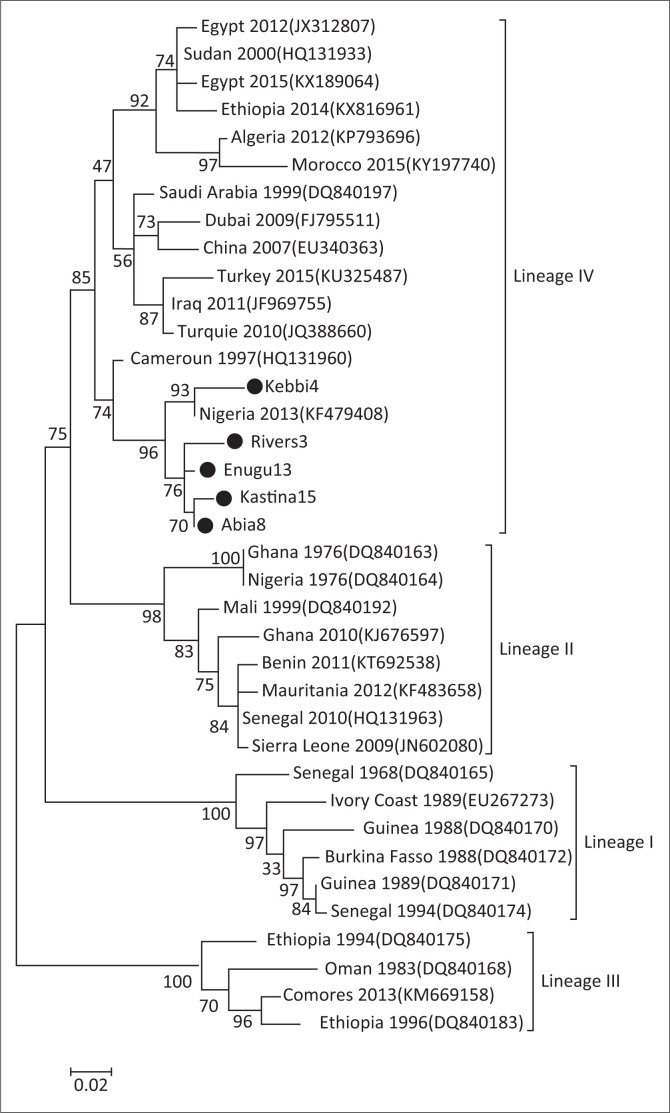
Phylogenetic tree of the partial N gene of Peste des petits ruminants viruses strains derived from samples in Nigeria and publicly available sequences. The sequences obtained in this study are indicated with a black dot. The numbers at the nodes are bootstrap values obtained from 1000 replicates are shown if > 50%.

**FIGURE 3:**
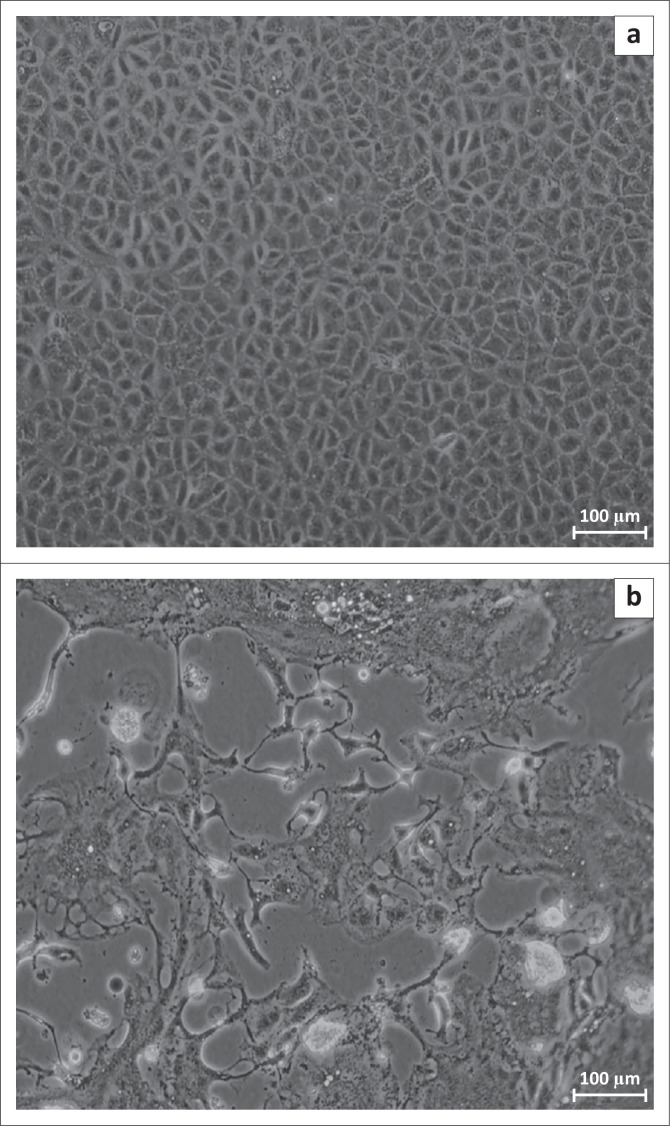
Canine histiocytic sarcoma cells at 100% confluence observed under a light microscope, ×100 magnification, when (a) non-infected or (b) with cytopathic effect (CPE) because of Peste des petits ruminants viruses infection.

Total nucleic acids were extracted from a total of 268 samples taken from 18 different states. Amplification of the target gene by RT-PCR using the pair of PPRV primers was successful for 73 samples (27%; Table 1). At least one sample was positive for PPRV of samples from 17 states: Plateau, Benue, Kwara, Bauchi, Adamawa, Taraba, Katsina, Kebbi, Enugu, Abia, Anambra, Oyo, Ondo, Osun, Rivers, Cross River and Akwa-Ibom. None of the samples were found to be positive amongst the 20 samples from Kano state (Table 1). Amongst the positive samples, 17 came from sheep (6%) and 55 from goats (21%). The sequence of the partial N gene was successfully obtained from one sample each collected from the states of Kebbi, Katsina, River, Enugu and Abia. Phylogenetic analysis showed that all five samples sequenced in this study belonged to lineage IV and clustered with a strain collected in Nigeria in 2013 (Figure 2; Woma et al. 2016) (R.E.4). The CPE was observed in only one out of the five samples used in the isolation attempt, which included the sample from the state of Kastina. The presence of PPRV in the cell culture was confirmed by RT-PCR (Figure 3).

## Discussion

Since they found that PPR as a disease in sheep and goats for the first time in Nigeria in 1973, and the eventual successful isolation of the virus in 1975 (Taylor & Abegunde 1979), the disease has remained a major threat for small ruminant production and has had negative impacts on food security, with PPR being the most economically important livestock disease in Nigeria.

Even though PPR has been of concern in the Nigerian livestock sector for a long time now, surprisingly few studies have been carried out to understand the molecular epidemiology of the disease in Nigeria. Additionally, the few studies that were conducted did not get as far as isolating and sequencing the isolates. Exceptions concern the virus detected and isolated in 1975 (Taylor & Abegunde 1979) and the molecular virus detection in 2002 (Shamaki 2002) that were found to cluster within lineage II. The results of this study provide evidence for the continued circulation of PPR across all six agro-ecological zones of Nigeria. The presence of PPRV was detected by N-gene-based RT-PCR in 27% (72/268) of random geo-spatially suspected samples, proving the circulation of PPRV in the study areas. Notably, we provide the first molecular confirmation of the presence of PPRV in the south-east agro-ecological zone.

In a similar previous study, a total of 35 samples out of 360 (9.7%) tested positive using RT-PCR, of which 25 were from oculo-nasal swabs and 10 were from tissue samples (Mantip et al. 2016). Similar previous studies using clinical cases in Ethiopia reported a positivity rate of 46.4% using RT-PCR (Alemu et al. 2019). In Morocco, a similar study reported a PPR positivity rate of 44.4% (16/36) using RT-PCR and a positivity rate of 80% in Sudan (Kwiatek et al. 2011). The presence of PPRV was also confirmed in 33.3% (7/21) and 51.2% (17/33) of clinical samples tested in Algeria and in north-central state in Nigeria, respectively, using a set of primers specific to the F gene of the PPRV (De Nardi et al. 2012). In northern and eastern Tanzania, the PPRV genome was also detected in 29.6% and 31.1% of the goats tested (Kgotlele et al. 2014). However, it has been shown that the level of positivity can be influenced by the type of sample used for diagnosis, the stage of infection and the type of gene targeted for RT-PCR (Luka et al. 2012). Such comparisons consequently only provide limited information to assess the RT-PCR technique or to provide details on the prevalence of the disease. Furthermore, as the data are provided by studies conducted in difficult field environments, 100% success is not feasible for the following reasons: (1) samples are not collected at the right time, as the locations of the outbreak are usually reached too late for antigen–gene detection, that is, the window for PCR diagnosis is too narrow; (2) according to these publications, the samples are stored differently: the shorter the harvest campaign, the faster the samples will be kept at the right temperature; (3) viral loads fluctuate depending on the host species and breed, as well as on the virulence of the PPRV strain, as demonstrated in experimental (Couacy-Hymann et al. 2007; Couacy-Hymann et al. 2009) and natural infections.

This study revealed a significantly higher rate of PPRV infection in goats than in sheep samples with RT-PCR, with 17 positive samples obtained from sheep (6%) compared with 55 (21%) from goats. The PPRV exhibits more or less similar levels of virulence in sheep and goats; however, goats are more severely affected, whilst sheep generally suffer from a milder form (Lefevre & Diallo 1990) or rarely suffer from a clinical disease (Fakri et al. 2017; El Hag Ali & Taylor 1984; Roeder et al. 1994). Nevertheless, high mortality rates were reported in sheep during an outbreak by Taylor (1984) who hypothesised that sheep possess innate resistance to the clinical effects of the disease; however, that occasional field strains can overcome this resistance resulting in high mortality rates (El Hag Ali & Taylor 1984). Breed may also affect the outcome of PPRV infection and its epidemiology; Guinean breeds (West African dwarf, Iogoon, Kindi and Djallonke) are known to be highly susceptible (Lefevre & Diallo 1990). British breeds exhibited severe clinical reactions when experimentally infected, whereas Sudanese breeds failed to develop a characteristic clinical response (El Hag Ali & Taylor 1984).

It has been suggested that PPRV be isolated from field samples in cell culture for further identification (FAO & OIE 2015; Lefevre et al. 1990). This study showed that the inoculation, isolation and propagation of PPRV in CHS-20 cells succeeded from the first passage of one of the selected positive samples, with the CPE characteristic in agreement with that described by the World Organisation for Animal Health (OIE 2019; Adombi et al. 2011). The presence of the virus in the CHS-20 medium was confirmed by collecting and testing the cell culture supernatant by RT-PCR.

Molecular characterisation of the circulating strains by phylogenetic analysis using the N gene is the most accurate way of identifying the lineage genetics belonging to new strains. This tool is important in understanding the epidemiology of PPRV and in tracking outbreaks in PPR-prone and endemic countries. Such information helps to establish the diversity and circulation of strains in the field, thereby tracing the space–time origin of PPRV and making it possible to estimate the risk of its introduction in the herd (Libeau et al. 2014). This study presents the first molecular confirmation of PPR infection in the states of Kebbi and Kastina (north-west), Abia and Enugu (south-east), and Rivers (south-south) in Nigeria. The sequences obtained showed that the samples tested belong to lineage IV. Previous studies showed that both lineages II and IV were circulating in neighbouring states in 2010-2012 (Mantip et al. 2016; Woma et al. 2016). Further sequencing of PPR-positive samples is needed to assess whether lineage II is still circulating in any of these five states.

The successful molecular findings and virus isolation in this study confirm the active presence of PPRV infections amongst populations of sheep and goats in all six agro-ecological zones, suggesting that PPR is currently endemic virtually everywhere in Nigeria. We, therefore, recommend systematic vaccination with thorough investigation of outbreaks and surveillance to contain outbreaks within the affected locations, states and regions of the country. We also recommend strengthening of the surveillance system, with emphasis on early detection, in epidemiologically closely linked administrative units (local government areas) to which the disease could potentially spread. Such interventions should be in line with broader regional and national control programmes for PPR in Africa.
